# “It helps me cope”: a meta-ethnographic synthesis on lived experiences of spiritual bypassing

**DOI:** 10.3389/fpsyg.2026.1852417

**Published:** 2026-08-03

**Authors:** Maitri Patel, Navin Kumar

**Affiliations:** School of Social Sciences and Languages, Vellore Institute of Technology, Vellore, Tamil Nadu, India

**Keywords:** meta-ethnography, qualitative synthesis, religious coping, spiritual bypass, spirituality and mental health, transpersonal psychology

## Abstract

Spiritual bypassing, referred to as the use of spiritual beliefs or practices to avoid unresolved psychological distress, is well represented in the etic (researcher- or outsider-derived) literature but has not previously been examined through a qualitative synthesis centered on lived experiences. This meta-ethnography addresses that gap. Using Noblit and Hare’s seven-phase approach alongside the eMERGe reporting guidance, five qualitative and mixed-methods studies conducted between 2014 and 2023 were synthesized, drawing on accounts from 55 participants across culturally and religiously diverse contexts. Participants from Muslim American, African American Pentecostal, predominantly Brazilian and Norwegian, African American addiction recovery, and Christian interpersonal recipient contexts were represented. The line-of-argument synthesis generated three third-order constructs: (1) The Emergence and Internal Dynamics of Spiritual Bypassing; (2) Social and Structural Mechanisms Sustaining Spiritual Bypassing; and (3) Resolution: From Defensive to Integrative Spirituality. The findings indicate that spiritual bypassing is more accurately understood not as a fixed psychological deficit located within the individual but as a contextually adaptive response to psychological pain, maintained by structural conditions and resolved through the differentiation from, rather than the abandonment of, spirituality. These results extend and, in several respects, challenge Pargament’s (2011) framework of religious coping and have implications for clinical practice and counseling education.

## 1 Introduction

Over the past three decades, spirituality has received growing research attention as one of the most fundamental dimensions of human experience, particularly in its relationship to psychological health. A substantial body of empirical evidence demonstrates that religious and spiritual engagement is associated with positive health outcomes, including reduced anxiety, greater resilience, and an enhanced capacity to find meaning in the face of suffering (Koenig, 2018; Pargament and Exline, 2022). However, not all forms of spiritual engagement support psychological wellbeing; some function to avoid it. One of the more clinically significant of these patterns is spiritual bypass.

The term was first introduced by transpersonal psychologist Welwood (1984), who used it to describe the tendency to draw on spiritual beliefs and practices as a way of sidestepping unresolved psychological pain rather than confronting it directly. Over subsequent decades, the concept was developed theoretically and empirically into a formally defined and measurable construct. Fox et al. (2017) define it as a defensive psychological posture in which spiritual beliefs or experiences are privileged over psychological needs, creating a means of avoiding difficult emotions. This definition has since informed both clinical frameworks for working with spiritually bypassing clients (Cashwell et al., 2007) and psychometric measurement, most notably through the development of the Spiritual Bypass Scale-13 (SBS-13; Fox et al., 2017), which has documented significant associations between spiritual bypass and depression, anxiety, and substance use.

It should be noted that even the use of such terms as “bypassing” implies a certain judgment regarding the use of spirituality as a way out of dealing with psychological suffering in a correct way. Although clinically relevant, this interpretation may obscure the fact that spirituality is indeed a tool for coping with pain that might be used by some people in the absence of other means. An emerging question in the literature concerns whether the term ‘spiritual bypassing’ adequately captures this more complex process, or whether it forecloses interpretive possibilities by framing as avoidance what may function, at least initially, as survival. Whether the construct warrants reconceptualisation is a question the present synthesis is positioned to address empirically, from the ground of lived experience.

Despite its clinical significance, theoretical development within the spiritual bypassing literature effectively stabilised by the early 2010s. Welwood (2000), Masters (2010), and Forman (2010) represent the terminus of substantive theoretical elaboration, and the development of the SBS-13 (Fox et al., 2017) constitutes the field’s sole advance in standardised measurement. Post-2017 scholarship has consisted primarily of empirical applications of the SBS-13 across varied populations (e.g., Fox et al., 2020) rather than conceptual or theoretical innovation. This plateau is itself significant as it may reflect the limits of what can be achieved from a purely etic (researcher- or outsider-derived) vantage point, one constituted almost entirely by clinicians and theorists positioned outside the lived experience of those who spiritually bypass. As a result, existing theories and measurement tools fail to adequately account for the lived experiences of those who experience spiritual bypass or who have received it from others. This positioning leads to the pathologizing of spiritual bypass as an individual defensive tendency, without considering the social values, religious power hierarchies, and sociocultural context within which it can emerge and be maintained.

Existing syntheses of the spirituality and mental health literature have predominantly taken quantitative forms, aggregating effect sizes and psychometric findings across large samples (Koenig, 2018; Pargament and Exline, 2022). Within the spiritual bypass literature specifically, scholarship has moved from theoretical elaboration to psychometric development to empirical application without at any stage undertaking a qualitative synthesis of first-person accounts. Reviews of religious coping have similarly favoured quantitative methods (Ano and Vasconcelles, 2005; Pargament et al., 1998), and no published qualitative evidence synthesis has examined lived experience of spiritual bypassing, religious avoidance, or related constructs as its primary focus. The emic (participant- or insider-derived) dimension of this phenomenon which is the meaning participants themselves assign to their own spiritual coping has therefore remained outside the scope of any systematic review.

A related and insufficiently examined question concerns the conditions under which spiritual engagement transitions from a defensive to an integrative function that is, from a mechanism of psychological avoidance to one capable of coexisting with emotional authenticity and sustained psychological engagement. This synthesis treats that trajectory as one of its central analytical concerns, foregrounding the endpoint of integrative spirituality as both a finding and a horizon for future research.

The present study addresses this gap by asking: *How is spiritual bypassing experienced, understood, and described in qualitative research, across psychological, cultural, and clinical contexts?*

## 2 Methodology

### 2.1 Epistemological position

This study is grounded in a social constructivist epistemological position. Social constructivism presupposes that knowledge and meaning are not found independently from human experiences but constructed through social, cultural, and historical interactions (Berger and Luckmann, 1966; Burr, 2015). Under the paradigm of social constructivism, each social phenomenon does not have one objective interpretation; instead, different social groups can develop unique but equally valid understandings about the phenomenon, depending on the conditions of their social relations and culture. The epistemology used in this study suits the nature of a synthesizing review focused on spiritual bypassing because the studied phenomenon cannot be generalized as having one universal definition, as its manifestations and underlying mechanisms differ depending on the context in which they take place.

This epistemological position is operationalised methodologically through meta-ethnography as developed by Noblit and Hare (1988), who explicitly characterised their approach as social constructivist in orientation (Noblit and Hare, 1988; Newman and Rashid, 2025). Meta-ethnography was selected because it is designed specifically for the interpretive synthesis of qualitative studies in which context is analytically central—making it the most appropriate method for a synthesis in which spiritual bypass is understood to be constituted differently across the Muslim American, African American Pentecostal, and addiction recovery contexts represented in the included studies (Britten et al., 2002; France et al., 2019).

A social constructivist epistemological position also entails a substantive reflexive obligation. Because knowledge is understood as produced rather than found, the researchers themselves are positioned subjects whose interpretive frameworks, disciplinary formations, and prior assumptions inevitably shape what they attend to in the data, which constructs they regard as plausible, and how they translate between studies. Reflexivity is accordingly treated in this study not as a procedural step applied retrospectively to a completed analysis, but as an ongoing epistemological commitment that runs through the entire synthesis process. The specific implications of the research team’s positioning for the interpretive choices made in this synthesis are examined in Section 2.5.

### 2.2 Search strategy and study identification

Searches were conducted across three primary databases of Scopus, ProQuest Dissertations and Theses Global, and Web of Science using three concept blocks combined with Boolean operators (AND between blocks; OR within blocks), as presented in full in Table 1. Concept block one captured the target construct and included the terms: *spiritual bypass*, *spiritual bypassing*, *spiritual avoidance*, and *premature transcendence*. Concept block two captured study design and included: *qualitative*, phenomenolog*, *lived experience*, interview*, *narrative*, *thematic analysis*, and *grounded theory*. Concept block three captured the broader domain and included: *spirituality*, *religion*, *transpersonal*, *counselling*, *psychotherapy*, and *coping*. No date restrictions were applied, acknowledging the limited and relatively recent development of the emic evidence base on this specific construct.

**Table 1:** Search strategies.

Database	Search strategy
Scopus	TITLE-ABS-KEY (“spiritual bypassing” OR “spiritual bypass”) AND TITLE-ABS-KEY (qualitative OR interview* OR phenomenolog* OR narrative* OR “thematic analysis”)
Web of Science (Social Sciences Citation Index—SSCI)	TS = (“spiritual bypassing” OR “spiritual bypass”) AND TS = (qualitative OR interview* OR phenomenolog* OR narrative* OR “thematic analysis”)
ProQuest Dissertations and Theses Global	(“Spiritual bypassing” OR “spiritual bypass”) AND (qualitative OR interview OR narrative OR phenomenology)
Google Scholar (Supplementary)	“Spiritual bypassing” qualitative; religion emotional avoidance qualitative (first 200 results screened by relevance)

Google Scholar was employed as an additional search resource, where the first 200 results were subjected to targeted phrase search screening *“spiritual bypassing qualitative.”* It is noted that the results returned by Google Scholar ranking algorithm may differ depending on the user’s profile and geographic location as well as on the search history and therefore cannot be seen as proof of the completeness of search coverage by this engine. Manual search was also conducted via references scanning from all the relevant articles discovered, as well as citation scanning from the key foundational sources (Welwood, 2000; Cashwell et al., 2007; Fox et al., 2017). No librarian or information specialist was involved in developing the search strategy; it is recognized as a shortcoming because the participation of specialists is now regarded as best practice in designing search strategies in qualitative evidence synthesis (Booth et al., 2016).

**Figure 1:**
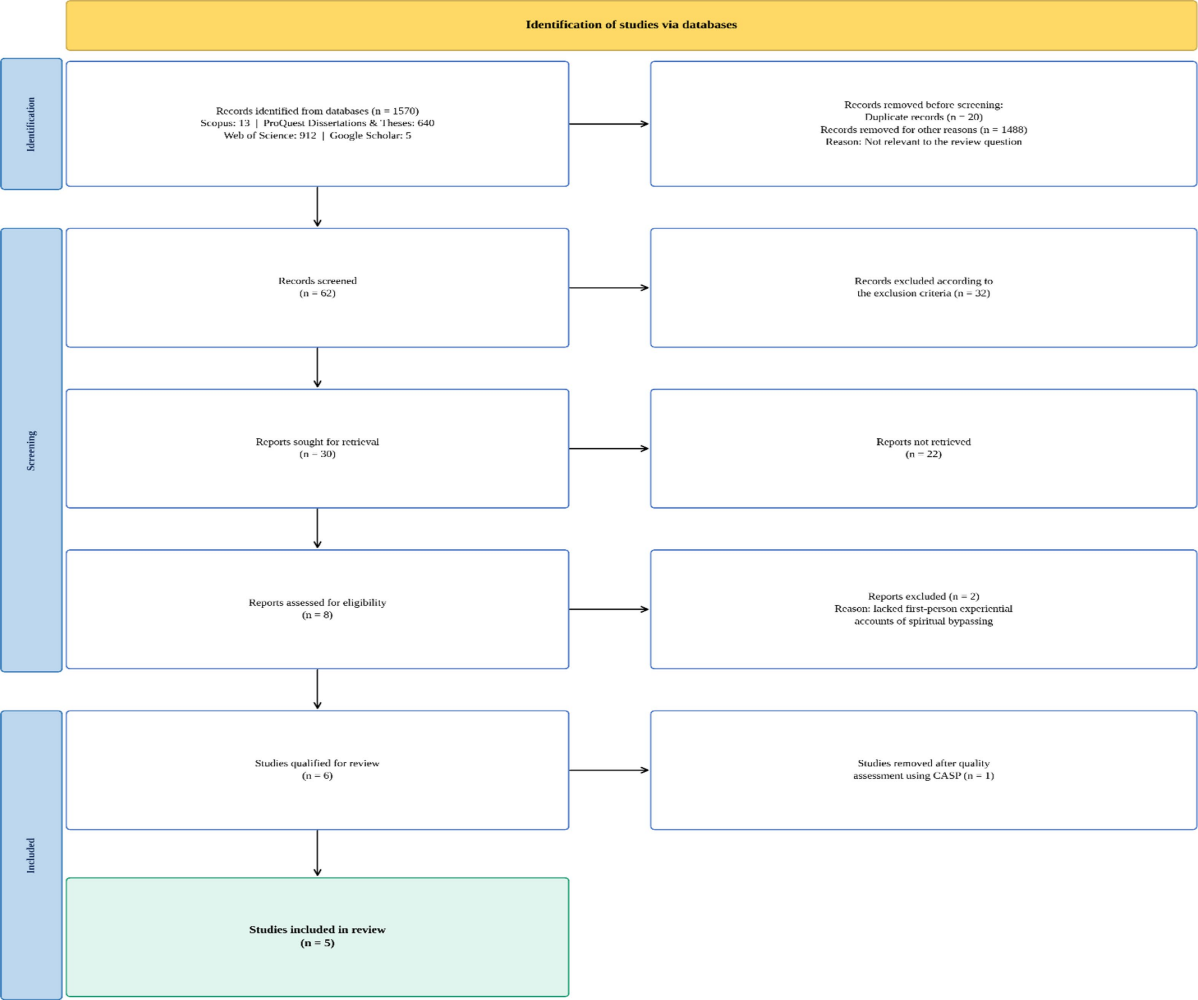
PRISMA 2020 flow diagram for the identification, screening, eligibility assessment, and inclusion of studies. One study, Galván (2025), was identified through manual searching but excluded following holistic quality appraisal on the grounds of four irreconcilable methodological concerns (see Section 2.4 and Table 2). Search reporting follows PRISMA 2020 (Page et al., 2021), supplemented by eMERGe (France et al., 2019) and ENTREQ (Tong et al., 2012).

The full selection process is illustrated in the PRISMA 2020 flowchart (Page et al., 2021; Figure 1). Search reporting adheres to PRISMA 2020 recommendations (Page et al., 2021), formulated initially for quantitative systematic reviews. Its application to a qualitative evidence synthesis is supplemented here by the eMERGe reporting guidance framework (France et al., 2019), which provides meta-ethnography-specific reporting standards, and is further informed by the Enhancing Transparency in Reporting the Synthesis of Qualitative Research (ENTREQ) statement (Tong et al., 2012), which was developed specifically to support transparent reporting in qualitative evidence syntheses.

### 2.3 Inclusion and exclusion criteria

Studies were included if they: (1) examined spiritual bypassing, or a clearly defined conceptual equivalent such as the use of spiritual practice to avoid unresolved psychological distress, or religious avoidance as a primary phenomenon of inquiry; (2) used qualitative or mixed-methods designs with an analytically separable qualitative component; (3) explored first-person experiential accounts through interviews, narratives, or reflective protocols; (4) were situated in a context in which the relationship between spirituality or religion and psychological wellbeing was a central concern of the research; (5) included adult participants aged 18 or above; and (6) were peer-reviewed journal articles or doctoral theses, published in English. No date restrictions were applied, as the emic evidence base on this specific construct is limited and relatively recent in its development (see Section 2.2 for rationale). The English language restriction, while operationally necessary, is acknowledged as a potential source of linguistic and geographic bias; this limitation is addressed in Section 5.

Studies were excluded if they: (1) were purely quantitative in design, or examined related constructs without direct reference to spiritual bypass or a clearly defined conceptual equivalent; (2) were theoretical, theological, or opinion-based without primary participant accounts; or (3) included children or adolescents, or relied exclusively on proxy accounts that is, accounts reported by someone other than the participant themselves (such as carer reports, clinician observations, or third-party narratives offered in lieu of participant voice) in the absence of direct participant data.

### 2.4 Quality appraisal

All identified studies were independently appraised using the Critical Appraisal Skills Programme (CASP) Qualitative Research Checklist (Long et al., 2020). Consistent with Long et al.'s (2020) explicit guidance that CASP is designed to structure holistic qualitative judgement rather than to generate numerical scores or composite ratings, each study was evaluated by both reviewers through narrative assessment across ten methodological domains. These domains addressed: (1) the clarity and appropriateness of the research aim; (2) the suitability of the qualitative methodology to the research question; (3) the appropriateness of the research design; (4) the rigour of the recruitment strategy; (5) the adequacy of data collection; (6) the transparency of the researcher–participant relationship; (7) the consideration of ethical issues; (8) the rigour of data analysis; (9) the clarity of the statement of findings; and (10) the research’s overall value and contribution.

Both reviewers conducted independent appraisals before convening to compare judgements. Where appraisals diverged, disagreements were resolved through structured discussion, return to the source study, and, where necessary, re-reading of relevant methodological sections. Quality appraisal disagreements were handled separately from the construct extraction process described in Section 2.5; no third party was required to resolve any appraisal disagreement. A holistic assessment of the trustworthiness of each study considered the reviewers’ overall opinion about the adequacy of methodological quality relative to the unique aims of this meta-analysis. None of the studies considered here was automatically excluded based on shortcomings in one dimension only.

Only one study by Galván (2025) was identified as lacking adequate methodological quality based on holistic consideration. These were: a discrepancy between the stated and actual research design; inconsistencies in the reported data collection method; unclear sample delimitation; and the conflation of theological advocacy with empirical research findings. No single reason was sufficient on its own but rather the overall and incompatible nature of these four reasons led to the exclusion of this study. Table 2 presents the holistic trustworthiness assessment for the five included studies.

**Table 2:** CASP appraisal summary for included studies.

CASP criterion	Gilbert-Reed (2023)	Herbin (2019)	Picciotto et al. (2017) †	Hamdan (2014) †	Rice (2023)
Q1. Clear qualitative aim?	✓	✓	✓	✓	✓
Q2. Qualitative methodology appropriate?	✓	✓	✓	✓	✓
Q3. Research design appropriate?	✓	✓	◑	✓	✓
Q4. Recruitment strategy appropriate?	◑	✓	◑	◑	✓
Q5. Data collection addressed question?	✓	✓	◑	✓	✓
Q6. Researcher–participant relationship?	◑	◑	◑	◑	◑
Q7. Ethical issues considered?	✓	✓	✓	✓	✓
Q8. Rigorous data analysis?	◑	◑	◑	◑	◑
Q9. Clear statement of findings?	✓	✓	✓	✓	✓
Q10. Research valuable?	✓	✓	◑	◑	◑
Holistic Trustworthiness Assessment	Moderate-to-high. Aim, methodology, and design clearly articulated. Partial ratings common in descriptive phenomenological dissertations. Included without caveats.	High. Strongest overall profile among included studies. Design, aim, and methodology clearly described and aligned. Included without caveats.	Moderate. Design mismatch; self-selected, geographically restricted sample; limited reflexivity. Retained for unique systematic account of bypass benefits and post-bypass categories. Lower-confidence contribution.	Moderate. Potential confirmatory risk from researcher-as-co-researcher positioning; personal network recruitment; educationally homogeneous sample. Retained for unique cultural contribution as sole study of Muslim Americans. Lower-confidence contribution.	Moderate-to-high. Well-executed explanatory sequential mixed-methods design. Quantitative findings used as contextualising evidence only (see §3.2.3).

Quality appraisal followed Long et al.’s (2020) guidance that CASP is designed to structure holistic qualitative judgement. ✓= Yes; ◑ = Partial; χ= No. † Picciotto et al. (2017) and Hamdan (2014) were retained as lower-confidence contributions; their findings are not cited as sole evidence for any interpretive claim.

### 2.5 Data extraction and synthesis procedure

The studies were synthesised using the seven-phase meta-ethnographic procedure outlined by Noblit and Hare (1988), with reporting guided by the eMERGe reporting guidance framework (France et al., 2019). The eMERGe framework was applied as a process-integrated reporting standard that informed how the synthesis was conducted and documented throughout, rather than as a checklist applied retrospectively to a completed write-up. Each study was read at least three times by the primary author: first to develop an overall understanding of the study and its epistemological positioning; second to identify first-order constructs defined as direct participant quotations drawn from study transcripts and results sections; and third to extract second-order constructs defined as the original researchers’ own interpretive themes and categories as reported in the results and discussion sections of each study. Second-order constructs were extracted verbatim as named by the original researchers and were not recast into the synthesisers’ own language at this stage, to preserve the integrity of the researchers’ own interpretive contributions.

The second reviewer independently repeated the full extraction procedure for all five studies before the two analysts convened to compare findings. Discrepancies were addressed through a structured two-stage process that differed depending on the type of disagreement. At the level of first- and second-order construct identification that is, disagreements about whether a given passage constituted a participant quotation or a researcher interpretation, resolution was achieved by returning to the relevant passage in the source manuscript and re-reading it in context until consensus was reached. At the level of interpretive translation that is, disagreements about how to characterise a second-order construct in relation to the emerging third-order synthesis, resolution involved extended discussion between the two reviewers, examination of other constructs from the same and adjacent studies, and, in all cases, return to the first-order constructs to verify that the proposed interpretation was grounded in participant voice. Disagreements at the interpretive level were more frequent than those at the identification level, reflecting the inherent ambiguity of translating across studies with differing epistemological frameworks and analytical vocabularies. In all instances, consensus was reached between the two reviewers without recourse to a third party.

The line-of-argument synthesis was selected as the mode of synthesis because the five included studies address the same broad phenomenon of spiritual bypassing from epistemologically and contextually distinct vantage points. Unlike reciprocal translation, which is used when studies are sufficiently comparable that their findings can be directly mapped on to one another, line-of-argument synthesis is appropriate when the included studies are not straightforwardly aggregable but can be used collectively to construct an overarching interpretive argument that accounts for the full range and diversity of findings, including their convergences, tensions, and contradictions (Campbell et al., 2003; Zimmer, 2006). The move from nineteen second-order constructs to three third-order constructs involved several iterative analytical steps. First, second-order constructs were arrayed across the five studies and compared for conceptual resonance identifying threads that ran across multiple study contexts as well as points of divergence or apparent contradiction. Second, the two reviewers engaged in structured interpretive discussion to identify the most parsimonious overarching argument that could account for the widest range of findings while remaining grounded in the first-order construct evidence. Third, provisional third-order constructs were named and then subjected to a verification pass: each was returned to the source data to confirm that the interpretive argument was demonstrably supported by participant voice and was not an artefact of the synthesisers’ theoretical preferences. Third-order constructs are accordingly treated throughout this paper as interpretive propositions developed from the data rather than as findings that emerge directly from any single included study.

The holistic quality appraisal findings described in Section 2.4 informed the weighting of evidence in the synthesis in two specific ways. First, findings from studies assessed as having stronger overall trustworthiness profiles particularly Herbin (2019) and Gilbert-Reed (2023), which received the most favourable holistic appraisals were treated as primary anchors for third-order construct development. Second, findings from studies assessed as having identifiable methodological limitations particularly Picciotto et al. (2017) and Hamdan, (2014) were incorporated into the synthesis but are not cited as sole evidential support for any interpretive claim. If a particular argument leans heavily on these two papers, it is addressed in the respective Results sections.

In the case of positionality of the authors, it merits elaboration rather than a mere disclosure. The main author has expertise in transpersonal psychology and a strong academic focus on the intersection of spirituality and psychological wellbeing. Transpersonal psychology as a tradition has historically maintained an affirming orientation toward spiritual development tending to frame spiritual experience as fundamentally growth-oriented, to conceptualise psychological suffering as a necessary precursor to spiritual advancement, and to read the arc of human development as moving toward greater integration of psychological and spiritual dimensions. These disciplinary tendencies may have shaped the primary analyst’s interpretive choices in specific and identifiable ways. The synthesis may have been inclined to foreground the adaptive dimensions of spiritual bypassing at onset reading the data through the lens of contextual necessity rather than examining it equally as possible harm and to read the trajectory of resolution as movement toward integration rather than entertaining equally grounded readings of the data that foreground ambivalence, harm, or irresolution. The Gilbert-Reed (2023) data, which documents interpersonally enacted bypass as coercion and spiritual weaponisation, is the study whose findings most clearly resist an adaptive framing; deliberate analytical attention was accordingly given to this data to avoid marginalising findings that challenge the synthesisers’ theoretical predispositions. These tendencies are identified here not as methodological failures but as potential sources of interpretive influence that mitigation strategies can reduce but cannot fully eliminate. Mitigation strategies comprised: an audit trail linking every synthesis claim to source texts; independent second review of all construct translations and third-order derivations by a researcher with a critical qualitative methodology background who does not hold a transpersonal psychology orientation; and the verification pass described above in which provisional third-order constructs were returned to participant voice before being accepted. Neither reviewer has direct personal experience of the specific forms of spiritual bypass documented in the included studies.

## 3 Results

Five studies were included in the synthesis, published between 2014 and 2023, charting the experiences of spiritual bypass across Muslim American, African American Pentecostal, predominantly Brazilian and Norwegian, African American addiction recovery, and Christian interpersonal recipient populations, with a total of 55 qualitative participants summarised in Table 3. A total of nineteen second-order constructs were identified alongside indicative first-order constructs. These nineteen are organised into three overarching third-order constructs developed through the line-of-argument synthesis: (1) The Emergence and Internal Dynamics of Spiritual Bypassing; (2) Social and Structural Mechanisms Sustaining Spiritual Bypassing; and (3) Resolution: From Defensive to Integrative Spirituality. These are summarised in Table 4.

**Table 3:** Summaries of the included studies.

Study (Author, Year, type)	Research design	Theoretical/Philosophical framework	Sample	Data collection	Data analysis	Key themes/Findings
Picciotto et al. (2017) Journal of Spirituality in Mental Health [Peer-reviewed article]	Qualitative Phenomenology (Conventional content analysis)	Phenomenological inquiry; emic perspective on lived experience of spiritual bypass	*N* = 8 4 female, 4 male 7 Brazilian, 1 Norwegian Ages 21–52 Mixed religious backgrounds: Catholic, Buddhist, Gnostic, Evangelical, SBNR	Semi-structured individual interviews (conducted online)	Conventional content analysis: codes per unit of meaning → categories → themes. Verification: reflexivity, investigator triangulation, peer debriefing	48 codes → 10 categories → 2 themes + 1 stand-alone category. Theme 1: Development of spiritual bypass (causes, natural stage, insight, resolving bypass). Theme 2: Effects of spiritual bypass (symptoms, negative consequences, benefits). Stand-alone: Prevention.
Hamdan (2014) Doctoral Dissertation Institute of Transpersonal Psychology [Unpublished dissertation]	Qualitative Heuristic inquiry (Moustakas, 1990)	Transpersonal psychology; holistic development framework; attachment theory (God-representation as secure/insecure attachment figure)	*N* = 10 5 women, 5 men Muslim Americans Recruitment: Muslim community organizations and networks.	Semi-structured individual interviews + Relationship Structures: Attachment Style Across Relationships Questionnaire	Heuristic analysis (Moustakas, 1990): immersion, incubation, illumination, explication, creative synthesis. Individual and composite depictions. Member-checking.	7 major themes including: relational emotional grief; relationship pain triggering return to religion; six dynamics of spiritual bypassing; eight remedies for overcoming spiritual bypassing.
Herbin (2019) Doctoral Dissertation Eastern University [Unpublished dissertation]	Qualitative Transcendental phenomenology (Moustakas, 1994)	Transcendental phenomenology; Africentric paradigm; Strong Black Woman construct; religious coping framework	*N* = 11 All African American Pentecostal women Eligibility: Scored positive on SBS-13 Ages 28–71 Recruitment: African American Pentecostal churches	Semi-structured interviews + Spiritual Bypass Scale-13 (participant screening)	Transcendental phenomenological analysis: epoché, phenomenological reduction, imaginative variation. 124 significant statements identified. Textural and structural descriptions.	6 themes: external spiritual forces at work during stress; providence of God as primary coping; Strong Black Woman persona supports self-negation; mental distress causes reassessment of standing with God; relief found in religious practices; culturally competent Christian therapists can assist.
Gilbert-Reed (2023) Doctoral Dissertation Liberty University [Unpublished dissertation]	Qualitative Descriptive phenomenology	Descriptive phenomenological analysis; Traumagenic Dynamics Model (Finkelhor and Browne, 1985); Sanctification Theory (Pargament and Mahoney, 2005)	*N* = 14 13 Caucasian, 1 African American 11 female, 3 male Ages 23–60 (M = 42, SD = 11.8) Christian context (multi-denominational)	Semi-structured individual interviews Recruitment: Facebook, Liberty Doctoral Commons Interview duration: 35–60 min	Descriptive phenomenological analysis: codes and sub-codes from transcripts, MAXQDA software, organised into 7 themes. Traumagenic Dynamics Model and Sanctification Theory applied post-analysis.	7 themes: misuse of spiritual authority; marginalisation of feelings; dismissal of personal challenges; misuse of scripture; lack of trust in self; distrust of religious institutions; intentional behaviour with unintentional outcomes.
Rice (2023) Doctoral Dissertation Eastern University [Unpublished dissertation]	Mixed methods Explanatory sequential design (Phase 1: quantitative survey; Phase 2: qualitative interviews; Phase 3: integration)	Explanatory sequential mixed methods (Creswell and Plano Clark); integrative psychospiritual framework; social identity theory	Phase 1 (Quantitative): *N* = 114 African Americans in opioid addiction recovery Phase 2 (Qualitative): *N* = 13 (purposive subsample from Phase 1, high SBS-13 scores)	Phase 1: Online surveys — SBS-13, BIAS Phase 2: Semi-structured individual interviews Phase 3: Joint display integration	Phase 1: ANOVA, t-tests, Pearson correlations, linear regression. Phase 2: Interpretive thematic analysis (hermeneutic circle). Phase 3: Joint display and narrative integration.	Quantitative: spiritualising and psychological avoidance positively associated with religious commitment; gender and income significant moderators. Qualitative 4 themes: self-awareness in recovery; conditions associated with wellness; conceptualising recovery with social identity; survival and resilience.

SBS-13, Spiritual Bypass Scale-13; BIAS, Belief Into Action Scale; SBNR, Spiritual but Not Religious; MAXQDA, qualitative data analysis software. All five studies were conducted in the United States.

**Table 4:** First-, second-, and third-order constructs.

Third-order construct	Second-order construct	First-order construct
The emergence and internal dynamics of spiritual bypassing	Picciotto et al. (2017) Causes of spiritual bypass	*“Trying to escape from reality, I found an escape route within spirituality, a way to flee my mundane life which appeared meaningless.”—Participant 5*
	Picciotto et al. (2017) Natural: stage, short-term coping strategy	*“I think I needed it, it was a part of my spiritual growth, it was a necessary stage, inevitable even. I had to live through that collapse and it made me wake up.”—Participant 2*
	Picciotto et al. (2017) Benefits of spiritual bypass	*“It was important to help me deal with pain — at least in the short term my religious practices would provide relief, would make me forget suffering.”—Participant 7*
	Hamdan (2014) Relational emotional grief	*“I reached an impasse in my life, a wall! I lost my job… and just when I thought that was bad, the same week I lost my job, my fiancé broke up with me. Tuesday I lost my job, Friday I lost my girl… I simply had no choice.”—Hamza*
	Hamdan (2014) Relationship pain triggers return to religion	*“I was going to mosque, like, Friday here and there. But when I got divorced, I had more alone time… I started going more to the mosque or tried to become a little bit more faithful.” —Alex*
	Herbin (2019) Relief found in religious practices	*“I’ve learned to trust God and depend on God and to wait on God… As long as I listen to God, the Lord will guide me through stressful times.”—Participant*
Social and structural mechanisms sustaining spiritual bypassing	Hamdan (2014) Six dynamics of spiritual bypassing	*“My spiritual practices were, at that time, very much a checklist type of practice. Like I had to do check, check, check. There wasn’t a deep connection of it and an understanding of why it’s so important.”—Omar*
	Herbin (2019) Strong Black Woman persona further heightened by denominational responsibilities	*“You came to church because you wanted to be in alignment with leadership… you put yourself on the back burner… You felt that if you would have said anything, you would have been rebuked and you would have been out of God’s will.”—Rachel*
	Herbin (2019) Mental distress causes reassessment of one’s standing with God	*“When my brain chemistry went out… I was so out of it that if you told me to pray, I forgot. Because my memory was gone inside of me spiritually, I wanted to pray but I could not remember the words.”—Participant*
	Gilbert-Reed (2023) Misuse of spiritual authority	*“It [spiritual bypass] was mostly from authority, spiritual authority… It was mostly from either the pastor or an associate pastor or an elder, which was like other people who have been deemed appropriate to make decisions for you.”—P3*
	Gilbert-Reed (2023) Misuse of scripture	*“It is like a weapon when it’s, I do not think, meant to be a weapon… I was around leaders that would manipulate any scripture to get what they wanted.”—P5*
	(Gilbert-Reed, 2023) Lack of trust in self	*“Speaking directly to sexuality, I think I’m at the place where to wrestle with what I believe and what I was taught, I still do not know, and I still have a confusion, and I still have shame.”—P1*
	Gilbert-Reed (2023) Intentional behaviour with unintentional outcomes	*“I think it [spiritual bypass] is intentional. I think if you let people have feelings, then you lose control. I think it’s always about control.”—P13*
Resolution: from defensive to integrative spirituality	Picciotto et al. (2017) Resolving spiritual bypass	*“First I started taking a certain drug and having psychological counseling to have conscience of my alienation. And it was then that I created the internal condition to take care of really looking at a bunch of stuff.”—Participant 2*
	Picciotto et al. (2017) Prevention	*“I recognized by reading that book from Robert Augustus Masters, realizing that spiritual teachers can have psychological issues, and also some of the readings of Ken Wilber were very helpful.”—Participant 8*
	Hamdan (2014) Intimate relationships as barometer of psychological growth	*“When you are alone, you have control over your emotions… when I enter into a relationship, my whole modus operandi changes… it ignites my issues that are sleeping and stirs up all my insecurities.”—Samar*
	Hamdan (2014) Integrating spirituality and psychotherapy for holistic development	*“I think it helps a lot more if the therapist understands your background and culture because they know where you are coming from. You spend less time teaching them and they are more focused on helping you.”—Hamza*
	Hamdan (2014) Eight remedies for overcoming spiritual bypassing	*“Practising Islam made me feel better, but only a little. When I was alone, I felt at peace and better, but then we would still fight… No matter how much I tried to tell myself that, it did not help.”—Omar*
	Herbin (2019) Culturally competent therapists who identify as Christian can assist in relieving stressful symptomatology	*“It’s okay to not be okay and that you can express that. That you are not going to go to Hell for expressing your mental state, how you feel.”—Participant*

First-order constructs are verbatim participant quotations drawn from included studies. Second-order constructs are the original researchers’ interpretive themes and categories. Third-order constructs are synthesiser-derived and represent the line-of-argument synthesis. Source attributions in brackets indicate the primary study from which each second-order construct was drawn.

### 3.1 The emergence and internal dynamics of spiritual bypassing

Across all five included studies, participants did not turn to spiritual practices as a deliberate strategy of avoidance. Rather, spiritual bypassing emerged as the nearest available response to pain often the only framework that an individual’s family history, community formation, or cultural heritage had equipped them to draw upon in moments of acute distress.

#### 3.1.1 Pain as the universal entry point

This sub-section draws on the second-order constructs of entry into spiritual practice through crisis (Hamdan, 2014), childhood integration of religion as a coping framework (Herbin, 2019), inflicted bypass through authority-mediated silencing (Gilbert-Reed, 2023), and addictive and racialised crisis as precipitating context (Rice, 2023). Taken across all five studies, the line-of-argument synthesis proposes that unbearable pain regardless of its form functions as the universal and consistent precipitant of spiritual bypass.

In all five studies, the entry point for spiritual bypass was an encounter with unbearable pain whether relational, structural, existential, or interpersonal that exceeded available coping resources. The form that this pain took varied between studies, but its role in precipitating spiritual bypass was consistent. Participants did not frame this turn as avoidance; they experienced it as the natural and often only plausible response to suffering. In Hamdan (2014), the entry point was characterised by the complete disorientation of the participants, who reported that they came to Islamic practice during professional and relational crisis with no other available framework for coping. In Herbin (2019), African American Pentecostal women described religion as deeply integrated into their lives from childhood. Spiritual practices served simultaneously as expressions of genuine faith and as mechanisms of emotional regulation, making it difficult to distinguish genuine religious engagement from what the clinical literature has begun to conceptualise as spiritual pain — a form of suffering arising when an individual’s spiritual framework itself becomes implicated in their psychological distress (Illueca et al., 2023) and the defensive use of that framework to contain it.

*“I reached an impasse in my life, a wall! I lost my job. and just when I thought that was bad, the same week I lost my job, my fiancé broke up with me. I’ll never forget that. Tuesday I lost my job, Friday I lost my girl. I simply had no choice. I mean what do you do, where do you go.” — Hamza* (Hamdan, 2014).

Additionally, the participants of Gilbert-Reed (2023) did not experience a self-initiated but rather an inflicted spiritual bypass, received from their religious and spiritual authority figures who, in their use of spirituality as a means of silencing emotional truth, provided a framework in which participants had no sanctioned space for acknowledging their own emotional realities. The entry point for participants of Rice (2023) was their addictive crisis, complicated further by their racialised circumstances, which made psychological realities both spiritually and materially costly.

#### 3.1.2 Spiritual bypassing as adaptive transitional scaffolding: an interpretive proposition

This sub-section draws on the second-order constructs of spiritual practice as crisis resource (Hamdan, 2014), the natural stage of bypass in spiritual development (Picciotto et al., 2017), and the Higher Power relationship as simultaneous resilience and avoidance mechanism (Rice, 2023). The line-of-argument synthesis proposes as an interpretive construction developed from these converging constructs rather than as a finding that emerges directly from any single study that spiritual bypass at onset may be understood as adaptive transitional scaffolding: an emergency resource that provided genuine containment at the moment of acute crisis, though one that was not designed to substitute permanently for psychological engagement.

At the moment when emotional pain was most acute and no alternative coping was available, spiritual practices provided genuine containment. Rice's (2023) participants described an equivalent function within the addiction recovery context: the Higher Power relationship provided both genuine resilience and a mechanism for bypassing psychological work, often simultaneously.

*“At the time, my faith, I would say definitely saved me. I really cannot imagine what I would have done without Islam that year. It was my anchor, especially in the beginning when I found out and I could not tell my family.” — Amira* (Hamdan, 2014).

The significance of this finding lies in its undermining of the predominant pathologization approach that is taken towards spiritual bypass within the body of research on this topic. It can now be seen as emergency scaffolding that was never intended to last forever. A better question which this synthesis highlights for the first time from an emic data set, is not whether bypass is occurring, but at what point in the developmental process it is occurring, and whether it is pre-empting or facilitating eventual psychological engagement.

#### 3.1.3 Short-term benefits reinforcing continued spiritual bypassing

This sub-section draws on the second-order constructs of benefits of spiritual bypass (Picciotto et al., 2017) and the description of spiritual practice as a drug whose effects wear off (Hamdan, 2014). The line-of-argument synthesis proposes that the continuation of spiritual bypass is contextually rational rather than individually pathological in the absence of equally immediate and accessible alternatives.

Picciotto et al. (2017) made the first systematic contribution to documenting the genuine benefits of spiritual bypassing, providing a dimension of the phenomenon that had received considerably less attention in prior etic accounts. Three categories of benefit were identified in that study: pain relief as a short-term coping mechanism; a sense of superiority defined by Picciotto et al. (2017) as “feeling more important or more advanced compared to those who do not practice spirituality,” and distinguished by those researchers from the healthy spiritual confidence that characterises sincere development, noting that “a confidence in one’s identity is a positive outcome of sincere spiritual development” which nonetheless provided identity stabilisation at moments of personal crisis; and the attenuation of existential meaninglessness.

*“It was important to help me deal with pain — at least in the short term my religious practices would provide relief, would make me forget suffering.”—Participant 7* (Picciotto et al., 2017).

Comparing this finding with Hamdan (2014) description of spiritual practice as a ‘drug whose effects eventually wear off’ suggests a process that may share structural features with other patterns of approach-avoidance, though the cross-study evidence base would need to be extended before this parallel could be drawn with confidence. The findings from the these two studies indicate that spiritual bypassing is a rational action if there are no other options available to deal with life circumstances.

### 3.2 Social and structural mechanisms sustaining spiritual bypassing

Findings from Herbin (2019), Gilbert-Reed (2023), and Rice (2023), with additional support from Picciotto et al. (2017), indicate that spiritual bypass is actively maintained by forces operating at community and structural levels. The line-of-argument synthesis proposes that the transition from onset to sustained spiritual bypass cannot be explained at the individual level: it is more accurately understood as a pattern reproduced through social structures that benefit from its continuation, a claim that these studies support independently from one another across three distinct structural contexts.

#### 3.2.1 Community scripts: the performance of spiritual strength

This sub-section draws on the second-order constructs of the Strong Black Woman schema and communal enforcement of emotional suppression identified in Herbin (2019). The line-of-argument synthesis proposes that community-level enforcement represents a qualitatively distinct sustaining mechanism from individual-level bypass, operating through collective identity norms rather than personal psychological dynamics.

Herbin (2019) study identified community-level enforcement among African American Pentecostal women through the construct of the ‘Strong Black Woman’ (SBW), in which emotional vulnerability was characterised as spiritual weakness, demonic vulnerability, or sin. Mental distress was interpreted as spiritual failure conflating what the clinical literature distinguishes as spiritual pain (Illueca et al., 2023) with moral or spiritual deficiency rather than as a psychological condition warranting professional support. This community response was produced through shared belief often embodied by both participants and community members alike, which reinforced spiritual bypassing behaviours not as a deliberate strategy but as the only culturally sanctioned response to psychological distress.

*“You came to church because you wanted to be in alignment with leadership. you put yourself on the back burner. You felt that if you would have said anything, you would have been rebuked and you would have been out of God’s will.” — Rachel* (Herbin, 2019).

#### 3.2.2 Interpersonal enforcement via religious authority

This sub-section draws on the second-order constructs of misuse of spiritual authority, misuse of scripture, lack of trust in self, and intentional behaviour with unintentional outcomes, all identified in Gilbert-Reed (2023). The line-of-argument synthesis proposes that interpersonally enacted bypass constitutes a structurally distinct and clinically more severe form of bypass than the self-generated patterns documented in other included studies, insofar as it involves one person’s agency actively constraining another’s psychological and emotional reality.

Gilbert-Reed (2023) documented that spiritual bypass is frequently imposed by authority figures such as pastors, elders, and other spiritual leaders who used religious frameworks to suppress, misuse, or abuse others’ emotional realities (Oakley et al., 2018). This was maintained through spiritual weaponisation, gaslighting, and the misuse of spiritual authority — a pattern that Gilbert-Reed’s participants consistently described as intentional on the part of those enacting it, even where those perpetrators believed themselves to be offering appropriate pastoral care.

*“I think it [spiritual bypass] is intentional. I think if you let people have feelings, then you lose control. I think it’s always about control.” - P13* (Gilbert-Reed, 2023).

Participants reported experiences of spiritual weaponisation in which biblical passages were invoked to dismiss suicidal ideations, enforce gender submission, or justify public shaming. The consequences of these experiences were enduring such as loss of self-esteem, heightened vigilance in religious environments, and a lasting cognitive dissonance between one’s authentic emotional responses and the framework of guilt and shame imposed by authority.

*“I feel like if I’m sitting in a church service, my guard is up. I’m questioning. I’m like ‘All of these people are liars, too.’”—P1* (Gilbert-Reed, 2023)*, on lasting hypervigilance following interpersonal spiritual bypass.*

#### 3.2.3 Structural factors: race, stigma, and hidden identity

This sub-section draws on the second-order constructs of hidden identity management and the intersection of racialised stigma with spiritual coping, both identified in Rice (2023). The line-of-argument synthesis proposes that structural conditions can make spiritual bypass not merely available but actively safer than psychological disclosure which is a dimension of spiritual bypass that no individually centred theory of the phenomenon can account for.

Rice (2023) extended the structural analysis beyond the religious community to encompass the broader conditions of racialised life in the United States. For African American participants navigating opioid addiction recovery, the combined stigma associated with being Black, addicted, and emotionally distressed produced what that study terms a ‘hidden identity’: the strategic management and concealment of multiple marginalised statuses to avoid further discrimination. Within this context, spiritual bypass was not only religiously sanctioned but structurally safer than psychological disclosure.

*“When I was in active addiction, I was homeless, so dealing with the stigma of being a Black male, but also being a homeless drug addict, it was kind of twofold. The reaction that I got from just people out on the street — people would clutch their purses close to them when I walked by.” - Mark* (Rice, 2023).

It is acknowledged that this synthesis is qualitative in method and that the following finding constitutes quantitative rather than qualitative evidence. It is included here not as primary synthesis evidence but as contextualising data drawn from the quantitative phase of a mixed-methods study included based on its qualitative component, and it is interpreted in relation to, rather than in place of, the qualitative findings. Rice's (2023) quantitative phase identified a statistically significant positive association between religious commitment and spiritual bypass scores (r = 0.589, *p* < 0.001), indicating that higher religious commitment was associated with higher bypass scores, moderated by gender and income. Taken together with the qualitative findings, this association suggests that bypass in this context was not simply a matter of individual religiosity but of structurally shaped coping under conditions of compounded stigma.

### 3.3 Resolution: from defensive to integrative spirituality

Across all five studies, resolution did not involve the abandonment of religious or spiritual life. Instead, it involved a qualitative shift from spirituality as armour against psychological pain to spirituality as a resource that could coexist with emotional authenticity and psychological work. This shift required a disruption to the self-sealing logic of bypass, followed by access to a relational or community context in which integration was genuinely possible.

#### 3.3.1 Relational disruption and embodied crisis as turning points

This sub-section draws on the second-order constructs of intimate relationships as a barometer of psychological growth (Hamdan, 2014), embodied crisis and breakdown of spiritual invulnerability (Herbin, 2019), and hitting ‘rock bottom’ in addiction recovery (Rice, 2023). The line-of-argument synthesis proposes that bypass persists as a self-reinforcing system until an experience external to its own logic as relational or embodied renders it legible to the person experiencing it, often for the first time.

Spiritual bypass persisted until an event or experience disrupted its self-reinforcing logic. Two types of disruption were identified. Most commonly described was relational disruption: intimate relationships that reactivated attachment wounds which spiritual practices could not reach. A second form was embodied crisis: the point at which spiritually bypassed psychological material exceeded what spiritual practice could contain.

*“When you are alone, you have control over your emotions. when I enter into a relationship, my whole modus operandi changes. it ignites my issues that are sleeping.” - Samar* (Hamdan, 2014).

Herbin (2019) documented cases in which severe mental distress, suicidal ideation, or ‘nervous breakdowns’ made it impossible to sustain the performance of spiritual invulnerability. Rice's (2023) participants described the equivalent as hitting ‘rock bottom.’ In both studies, the crisis was experienced not as spiritual failure but as the point at which bypass became legible to the person experiencing it, often for the first time.

#### 3.3.2 Catalyst: the trusted, culturally legible other

This sub-section draws on the second-order constructs of integrating spirituality and psychotherapy for holistic development (Hamdan, 2014), culturally competent Christian therapists as sources of relief (Herbin, 2019), and conditions associated with wellness in recovery (Rice, 2023). According to the line-of-argument synthesis, the resolution process is mainly characterized by relational and culture-related processes and insights other than the individual’s psychological realization alone, and fluency in the cultural context is crucial to achieving this resolution.

In the literature from Hamdan (2014), Herbin (2019), and Rice (2023), the breakthrough occurred as a result of an alternate perspective on the individual’s circumstances by someone trusted such as a friend, therapist, or member of the community that did not require the person to make a choice between her spirituality and psychological well-being. What distinguished the catalyst was its connection to the individual’s spiritual world, whether in the form of someone from the same spiritual context or someone familiar with the religious beliefs involved. Those who sought assistance from non-religious counselors had to spend more time informing the therapist about Islam.

*“I think it helps a lot more if the therapist understands your background and culture because they know where you are coming from. You spend less time teaching them and they are more focused on helping you.” - Hamza* (Hamdan, 2014).

This synthesis indicates the importance of cultural fluency in a therapeutic setting, facilitating meaningful psychological engagement while allowing individuals to maintain their spiritual identities.

#### 3.3.3 Integration as the endpoint of resolution

This sub-section draws on the second-order constructs of eight remedies for overcoming spiritual bypassing and the role of intimate relationships as a developmental barometer (Hamdan, 2014); post-spiritual bypass spirituality categories (Picciotto et al., 2017); and the relational experience of being permitted emotional distress without it being interpreted as spiritual deficiency (Herbin, 2019; Rice, 2023). The line-of-argument synthesis proposes as its most clinically significant interpretive conclusion that integration, rather than abandonment of spiritual life, constitutes the demonstrable endpoint of bypass resolution across all five included studies.

Resolution culminated not in a rejection of spirituality but in its integration with psychological self-awareness. Hamdan (2014) eight remedies including psychotherapy, assumed responsibility for emotional experience, recognition of non-linear growth, tolerance of emotional ambivalence, and a multi-tool coping repertoire representing the most systematically articulated account of this integration in the included literature. Herbin (2019) and Rice (2023) located integration in the relational domain: the experience of being permitted, within a therapeutic or community context, to be distressed without that distress being immediately recorded as spiritual deficiency.

*“It’s okay to not be okay and that you can express that. That you are not going to go to Hell for expressing your mental state, how you feel.” - Participant* (Herbin, 2019), *articulating the permission that initiates resolution.*

Picciotto et al.'s (2017) post-spiritual bypass categories provide the clearest phenomenological articulation of this endpoint, describing forms of spirituality able to hold both transcendence and emotional pain simultaneously and distinguishing this post-bypass integration from both the unreflective religious practice that preceded bypass onset and the bypass period itself.

## 4 Discussion

This is, to our knowledge, the first meta-ethnographic synthesis of qualitative evidence on the lived experience of spiritual bypass. The line-of-argument synthesis generated an interpretive argument that no single included study could have produced: that spiritual bypass is best understood not as a fixed psychological deficit located within the individual but as a contextually adaptive response to unbearable pain that is subsequently sustained by structural forces and is resolved through the differentiation, not the abandonment, of spiritual life. This argument extends and, in several respects, challenges the conceptual architecture of the existing bypass literature. Three implications are developed below.

### 4.1 Extending Pargament’s framework: spiritual bypass as temporally evolving religious coping

The most generative dialogue to emerge from this synthesis is with Pargament's (2011) framework of religious coping. Positive and negative religious coping behaviors differ greatly in nature, as described by Pargament. For example, positive religious coping techniques include the use of benevolent religious reframing, collaboration with God to solve problems, and congregational assistance, which involves actively engaging with adversity using spiritual resources. Examples of negative religious coping behaviors include spiritual struggles, feelings of punishment from God, and using religion as an escape from adverse situations.

Extending Pargament’s original distinction, spiritual bypass at onset resembles positive religious coping by providing effective containment, relief, and a feeling of community inclusion. Over time, however, continuing the strategy of spiritual bypass as a method of escaping any necessary healing processes, such as grieving or processing difficult experiences, can result in psychological stasis while spiritual practice continues and progresses. The synthesis findings indicate that this shift is not hypothetical but constitutes the central developmental arc documented across all five included studies.

The challenge is that existing instruments such as the SBS-13 do not account for this developmental shift. The scale captures the degree to which someone is relying on spiritual practice at a given moment without distinguishing whether that reliance reflects an adaptive early-stage response to crisis or a more entrenched pattern of avoidance therefore, from the instrument’s perspective, the two phenomena are indistinguishable. Such an observation is confirmed by Rice's (2023) results: higher religious commitment was positively associated with scores on the SBS-13, but high religious commitment in crisis conditions is not indicative of spiritual bypassing. It may simply reflect people drawing on the resources their faith tradition has equipped them to use. The scale, as currently constructed, cannot make that distinction.

The synthesis findings therefore suggest a need for a more sensitive instrument, one that would ask not only how much someone is relying on their spirituality, but at what point in their experience and in relation to what circumstances, distinguishing a faith response that is helping someone survive a crisis from one that has become a long-term substitute for psychological growth.

Drawing directly from the first- and second-order constructs identified in this synthesis, such a revised or supplementary measure would need to capture at minimum three dimensions not addressed by the SBS-13. First, a *temporal dimension*: distinguishing whether spiritual reliance is occurring at an acute crisis point where elevated coping may be adaptive from a sustained long-term pattern in which spiritual practice has become a substitute for psychological engagement. Second, a *source dimension*: distinguishing between personal bypass, community bypass implemented through collective norms like the Strong Black Woman framework (Herbin, 2019), and interpersonal imposition of bypass performed by people with authority over others (Gilbert-Reed, 2023). SBS-13 cannot distinguish a person who is bypassing from the person who is having bypass actively imposed upon them, even though these represent clinically and ethically distinct situations. Third, a *directionality dimension:* considering if spiritual practice happens in place of psychological engagement with the problem presented. Instruments that deal with concepts related to bypass include Brief RCOPE (Pargament et al., 1998) and Religious and Spiritual Struggles Scale (Exline et al., 2014), which partially lay the foundation, but none of the two measures were developed to capture temporal and source dimensions as highlighted by this synthesis.

### 4.2 Challenging the individualising tendency of the etic literature

A second important finding challenges a foundational assumption in the spiritual bypass literature that bypass is primarily a personal problem rooted in an individual’s psychological tendencies, defensive habits, or unresolved developmental history. The present synthesis indicates that for many of the people most affected by spiritual bypass, this framing does not hold. It is not simply incomplete; it can actively obscure what is going on.

For African American Pentecostal women in Herbin (2019), the expectation of emotional strength operates not as an informal social preference but as an integral element of the cultural identity of the Strong Black Woman, meaning that disclosing psychological distress jeopardises one’s standing as a woman of faith in the community. The findings of Gilbert-Reed (2023) further indicate that spiritual bypass, when enacted interpersonally, was not driven by conscious harmful intent; rather, those who perpetrated it believed they were offering appropriate spiritual care, a perception shaped not by individual malice but by the institutional and doctrinal frameworks within which their understanding of compassionate care had been formed.

The evidence presented in this synthesis further indicates that spiritual bypassing is shaped by structural factors that lie largely outside the individual including racial mistrust, surveillance, and institutional discrimination (Rice, 2023). Acknowledging psychological distress carries genuine material risks in these contexts, and no level of personal motivation or psychological insight can simply override structural barriers. There is therefore a need for community-level strategies targeting the institutional frameworks within which spiritual bypassing is produced and sustained, alongside individual-level clinical interventions. This is consistent with the critical psychotherapy literature, which indicates that psychological distress cannot be treated in structural isolation (Morrow, 2007).

An important counterargument to the adaptive framing developed in this synthesis deserves direct engagement. The argument that spiritual bypass is contextually adaptive which is well-evidenced for self-generated bypass in the contexts of Hamdan (2014) and Picciotto et al. (2017) risks naturalising dynamics that, in other contexts, constitute harm rather than adaptation. The interpersonal bypass documented in Gilbert-Reed (2023) including the weaponisation of scripture, spiritual gaslighting, and the enforcement of gender submission is not an individual’s adaptive response to pain but an experience of coercion from which participants suffered enduringly and from which no adaptive logic protected them. Imposing an adaptive frame on these dynamics risks minimising harm that the spiritual abuse literature (Oakley et al., 2018) is designed to name precisely. The synthesisers accordingly acknowledge that the adaptive interpretation is most clearly supported by the self-generated bypass data, and that future analyses of interpersonally enacted bypass may be better served by frameworks drawn from trauma and religious abuse research than from the coping tradition.

### 4.3 Resolution as psycho-spiritual differentiation: implications for transpersonal practice

The pattern that participants resolve spiritual bypass by differentiating rather than abandoning their spirituality resonates with a central claim within the transpersonal psychology tradition that psychological and spiritual development are deeply interrelated and mutually conditioning processes (Wilber, 2000; Ferrer, 2002). It is important to note, however, that both Wilber and Ferrer write as theorists within the transpersonal tradition rather than as empirical researchers, and their frameworks represent theoretical positions rather than established empirical findings. Some frameworks within this tradition understand the human person as tripartite- encompassing body, mind, and spirit as distinct though interrelated dimensions (Ferrer, 2002), and on this view the psychological and spiritual are distinguishable even where they are mutually conditioning. The convergence between the theoretical claim and the emic data presented here is accordingly noted as theoretically suggestive rather than as mutual validation; the two forms of evidence are not epistemologically equivalent, and their resonance does not constitute corroboration.

The present synthesis finds a pattern consistent with what Picciotto et al. (2017) first proposed at the single-study level that spiritual bypass can function as a natural and necessary stage in a person’s spiritual development by identifying the same developmental arc across five studies. This is not to confirm that bypass is a universal stage, but to suggest that, within the contexts documented here, bypass is more accurately read as a stage to be moved through than as a problem to be simply corrected. As Hamdan (2014) eight remedies illustrate, resolution does not mean leaving spirituality behind. What it achieves is a mode of spiritual engagement that coexists with genuine psychological self-knowledge, accountability in relationships, and the capacity to tolerate difficult emotions. This endpoint which this synthesis terms integrative spirituality merits dedicated empirical investigation in its own right.

The available evidence indicates that bypass resolution involves not the replacement of spiritual practice with psychological work but a qualitative shift in how spiritual resources are held — from a defence against emotional experience to a medium through which emotional experience can be tolerated, processed, and given meaning. The factors, contexts and behaviors associated with making this shift are yet empirically undefined. Future instrument development may also benefit from operationalising integrative spirituality as a measurable endpoint rather than treating the mere reduction of bypass scores as an adequate index of resolution.

Furthermore, the cultural and religious fluency that the synthesis identifies as a constitutive condition of resolution carries significant implications for clinical training. Contemporary multicultural counselling competency frameworks, including the Multicultural and Social Justice Counseling Competencies (Ratts et al., 2016) and the APA Multicultural Guidelines (American Psychological Association, 2017), have advanced considerably since the foundational work of Sue et al. (1992). Nevertheless, neither current framework adequately addresses the specific intersection of spiritual identity, community-level bypass enforcement, and the clinical recognition of interpersonally imposed spiritual harm. Appropriate training modules must therefore be developed to equip practitioners with the knowledge and skills to navigate these dynamics competently.

The clinical implications of this synthesis appear to extend beyond the individual psychotherapy setting. The populations most affected by spiritual bypass as documented here- African American women navigating Pentecostal community norms, individuals in opioid addiction recovery, and recipients of interpersonal bypass from religious authority, are based on the contexts documented across the included studies, more likely to first encounter behavioural health support through integrated primary care, community health centres, or faith-based organisations than through private psychotherapy. The biopsychosocial-spiritual model (Sulmasy, 2002), which positions spiritual wellbeing alongside biological and psychological dimensions as integral to clinical assessment, provides a directly applicable framework for clinicians in these settings. Operationalised within the time constraints of a primary care encounter, cultural fluency with a patient’s religious framework does not require extended exploration; it requires the clinician’s capacity to recognise when spiritual language is functioning as avoidance of psychological disclosure, and to hold open rather than foreclose the psychological dimension in their response.

## 5 Limitations and directions for future research

Several limitations constrain the interpretation of these findings. First, the epistemological diversity among the included studies introduces a level of incommensurability that line-of-argument synthesis can only partially resolve. The included studies span heuristic inquiry, transcendental phenomenology, descriptive phenomenology, conventional content analysis, and explanatory sequential mixed methods epistemological frameworks that carry different assumptions about the nature of knowledge, the status of the researcher, and the role of interpretation. While line-of-argument synthesis is specifically designed to accommodate such diversity, its capacity to do so without residual interpretive loss is inherently limited, and readers should hold the third-order constructs as provisional interpretive propositions rather than as definitive aggregations of convergent evidence.

Second, all five studies rely on retrospective accounts from participants who had, to varying degrees, already moved beyond their experiences of spiritual bypass. This reliance on retrospective reporting introduces the possibility of recall bias, which may influence how participants reconstruct both the onset and the resolution of bypass. Retrospective narration of resolved experience tends to impose coherence, a sense of developmental arc or necessary progression on what may have been, at the time of occurrence, a more ambiguous and non-linear process. Additionally, the absence of participants who had not resolved their experiences of spiritual bypass biases the third construct toward successful outcomes, thereby limiting the generalisability of the proposed pathway of resolution. Future research should include participants at different points in the bypass trajectory, including those who are actively bypassing and those for whom bypass has remained chronic and unresolved.

Third, the geographical and cultural scope of the available evidence is restricted. All five studies were conducted in the United States, and none examines spiritual bypass within non-Anglophone or non-Western contexts. While the synthesis represents meaningful cultural diversity within the United States across Muslim American, African American Pentecostal, and addiction recovery communities as this diversity is intra-national rather than cross-national in scope. Such claims about cross-cultural generalizability should be understood within this context. In addition, the absence of several significant religions such as Hinduism, Buddhism, Judaism, Sikhs, and even a selection of secular spirituality practices further highlights that there are important gaps within the literature used for this study. The three third-order constructs that have been identified have been identified through an examination of the data from a limited cultural perspective, and therefore it is possible that the findings cannot be generalized to other cultural settings where the structure of the relationships discussed are different.

Fourth, all included studies were reported in the English language. While such a constraint is a necessity, it creates a possible bias of a geographical and linguistic nature: any qualitative study written in another language that is not available in English is automatically excluded from this synthesis. This aspect becomes crucial in light of the fact that one of the key gaps identified in this synthesis was that there was a lack of non-Western evidence, and it might well be the case that a number of relevant studies were conducted in South Asia, East Asia, or Latin America and reported in another language. Newman and Rashid (2025) who perform a similar meta-ethnography also recognize the possibility of exclusion of an unknown amount of data because of the English language constraint.

Fifth, the quality appraisal process, although carried out systematically in accordance with the Long et al. (2020) recommendations regarding the application of CASP as a tool for making judgment calls, is an inherently subjective one regarding assessment of methodology. There are still significant debates about the methodology of qualitative evidence synthesis itself (Long et al., 2020), and the decisions to include and weigh evidence discussed here were made as such rather than objectively established. For example, the decision to keep Picciotto et al. (2017) and Hamdan (2014) as lower-level sources despite certain issues with sample representativeness and researcher bias is based on the idea that their theoretical contribution outweighs any methodological drawbacks. The readers are advised to consider the holistic judgments of trustworthiness offered in Table 2 when assessing the relative weight of evidence for each of the third-order constructs.

Sixth, the background of the primary researcher in transpersonal psychology could be viewed as a potentially influential factor which would require the application of mitigation measures from Section 2.5. As noted in that section, transpersonal psychology as a tradition has historically tended toward affirming spiritual development and framing resolution as integrative, and these tendencies may have inclined the synthesis toward foregrounding adaptive interpretations of bypass at the expense of equally grounded readings that foreground harm, ambivalence, or irresolution. Readers are encouraged to hold the adaptive framing of the synthesis in productive tension with the counterargument developed in Section 4.2 that for the populations documented in Gilbert-Reed (2023), bypass is more accurately read through frameworks of coercion and harm than through frameworks of coping and adaptation.

Seventh, this synthesis is subject to the standard risk of publication bias in qualitative evidence synthesis. Qualitative studies documenting more ambivalent, harmful, or irresolvable experiences of spiritual bypassing may be less likely to have been submitted to peer-reviewed journals, accepted for publication, or indexed in the searchable databases used in this review. The available emic literature may consequently over-represent accounts in which participants achieved some degree of resolution, a pattern that is itself consistent with the cross-study trend toward resolution observed across all five included studies, which is named here as a limitation of the third third-order construct rather than a straightforward finding. The absence of published qualitative studies of individuals who are chronically bypassing without resolution is therefore difficult to interpret: it may reflect a genuine rarity of such experiences, or it may reflect the structural conditions under which qualitative evidence about spiritual bypass reaches publication.

Future research should prioritise seven areas. First, *longitudinal studies* tracking the bypass trajectory prospectively from onset through resolution - prospective designs would address the recall bias limitation identified above and would allow the developmental arc proposed in this synthesis to be tested against temporally immediate participant accounts. Second, *non-Western and non-Anglophone contexts* studies of spiritual bypass within Hindu, Buddhist, Jewish, Sikh, and secular spiritual traditions, and within languages other than English, are urgently needed to determine whether the three third-order constructs hold outside the United States context. Third, *chronic bypass without resolution* namely, a study that focuses on people who are currently bypassing and have not yet resolved their issues will help to eliminate the survivorship bias present in the current literature. Fourth, *intervention studies involving community-level and pastoral formation* that would test the structural hypotheses in real-world settings while the review concludes community enforcement to be a sustainable force in this context, there are currently no intervention studies that look at how change on a community level within the religious context might affect such enforcement practices. Fifth, *revision of the SBS-13* considering the emic evidence presented in Section 4.1, specifically to incorporate temporal, source, and directionality dimensions not captured by the current instrument. Sixth, empirical study focused on integrative spirituality as an outcome of bypass resolution, while there is considerable theory about how to go from one form to another, this process is empirically unexamined. Studies examining these conditions, including both therapeutic and community-based contexts, would constitute the most clinically actionable extension of this synthesis. Seventh, *instrument development* operationalising integrative spirituality as a distinct, measurable endpoint, rather than treating reduced bypass scores as an adequate proxy for resolution such an instrument would need to capture both the qualitative transformation in how spiritual resources are held and the individual’s capacity to tolerate emotional experience within, rather than instead of, their spiritual engagement.

More broadly, this synthesis suggests that meta-ethnography represents an underutilised methodological resource in the study of spirituality and mental health. As the counselling, clinical psychology, and pastoral care literatures give increasing attention to the integration of spirituality and religion into practice, the first-person evidence base produced by this synthesis provides a necessary grounding in the experience of those for whom bypass is not a theoretical construct but a lived reality, one with consequences for psychological development, relational health, and, in some cases, survival.

## Data Availability

The original contributions presented in the study are included in the article/supplementary material, further inquiries can be directed to the corresponding author.

## References

[ref1] American Psychological Association (2017). Multicultural Guidelines: An Ecological Approach to Context, Identity, and Intersectionality. Washington: American Psychological Association.

[ref2] Ano G. G. Vasconcelles E. B. (2005). Religious coping and psychological adjustment to stress: a meta-analysis. J. Clin. Psychol. 61, 461–480. doi: 10.1002/jclp.20049, 15503316

[ref5] Berger P. L. Luckmann T. (1966). The Social Construction of Reality: A Treatise in the Sociology of Knowledge. New York: Anchor Books.

[ref6] Booth A. Sutton A. Papaioannou D. (2016). Systematic Approaches to a Successful Literature Search. 2nd Edn London: Sage.

[ref7] Britten N. Campbell R. Pope C. Donovan J. Morgan M. Pill R. (2002). Using Meta-ethnography to synthesise qualitative research: a worked example. J. Health Serv. Res. Policy 7, 209–215. doi: 10.1258/135581902320432732, 12425780

[ref8] Burr V. (2015). Social Constructionism. 3rd Edn London: Routledge.

[ref9] Campbell R. Pound P. Pope C. Britten N. Pill R. Morgan M. . (2003). Evaluating Meta-ethnography: a synthesis of qualitative research on lay experiences of diabetes and diabetes care. Soc. Sci. Med. 56, 671–684. doi: 10.1016/S0277-9536(02)00064-3, 12560003

[ref10] Cashwell C. S. Bentley P. B. Yarborough J. P. (2007). The only way out is through: the peril of spiritual bypass. Counsel. Values 51, 139–148. doi: 10.1002/j.2161-007x.2007.tb00071.x

[ref13] Exline J. J. Pargament K. I. Grubbs J. B. Yali A. M. (2014). The religious and spiritual struggles scale: development and initial validation. Psychol. Relig. Spiritual. 6, 208–222. doi: 10.1037/a0036465

[ref14] Ferrer J. N. (2002). Revisioning Transpersonal Theory: A Participatory Vision of Human Spirituality. Albany: State University of New York Press.

[ref500] Finkelhor D. Browne A. (1985). The traumatic impact of child sexual abuse: a conceptualization. Am. J. Orthopsychiatry. 55, 530–541. doi: 10.1111/j.1939-0025.1985.tb02703.x 4073225

[ref15] Forman M. D. (2010). A Guide to Integral Psychotherapy: Complexity, Integration, and Spirituality in Practice. Albany: State University of New York Press.

[ref16] Fox J. Cashwell C. S. Picciotto G. (2017). The opiate of the masses: measuring spiritual bypass and its relationship to spirituality, religiosity, depression, anxiety, and substance use. Spirit. Clin. Pract. 4, 274–287.

[ref17] Fox J. Picciotto G. Cashwell C. S. Worthington E. L. Basso M. J. Brow Corrigan S. . (2020). Religious commitment, spirituality, and attitudes toward god as related to psychological and medical help-seeking: the role of spiritual bypass. Spirit. Clin. Pract. 7, 178–196. doi: 10.1037/scp0000216

[ref18] France E. F. Cunningham M. Ring N. Uny I. Duncan E. A. S. Jepson R. G. . (2019). Improving reporting of meta-ethnography: the eMERGe reporting guidance. BMC Med. Res. Methodol. 19, 1–19. doi: 10.1186/s12874-018-0600-0, 30709371 PMC6359764

[ref19] Galván M. (2025) “Spiritual Bypass among Christian Women in Charismatic Communities: A Phenomenological Study.” [Unpublished Doctoral Dissertation]

[ref20] Gilbert-Reed P. (2023). Wounding Words: A Phenomenological Study of How Individuals Experience Receiving Spiritual Bypass. [Doctoral Dissertation]. Lynchburg, VA: Liberty University.

[ref22] Hamdan K. (2014). Spiritual Bypassing: Unveiling Dynamics and Remedies of It among Muslim American Participants.” [Doctoral Dissertation]. Palo Alto, CA: The Institute of Transpersonal Psychology.

[ref23] Herbin A. P. (2019). The Subsistence of Spiritual Bypass among African American Pentecostal Women through their Understanding and Expressions of their Faith.” [Doctoral Dissertation]. St. Davids, PA: Eastern University.

[ref24] Illueca M. Bradshaw Y. S. Carr D. B. (2023). Spiritual pain: a symptom in search of a clinical definition. J. Relig. Health 62, 1920–1932. doi: 10.1007/s10943-022-01645-y, 36083524 PMC9461389

[ref25] Koenig H. G. (2018). Religion and Mental Health: Research and Clinical Applications. San Diego: Academic Press.

[ref27] Long H. A. French D. P. Brooks J. M. (2020). Optimising the value of the critical appraisal skills Programme (CASP) tool for quality appraisal in qualitative evidence synthesis. Res. Methods Med. Health Sci. 1, 31–42. doi: 10.1177/2632084320947559

[ref28] Masters R. A. (2010). Spiritual Bypassing: When Spirituality Disconnects Us from What Really Matters. Berkeley: North Atlantic Books.

[ref29] Morrow S. L. (2007). Qualitative research in counseling psychology: conceptual foundations. Counsel. Psychol. 35, 209–235.

[ref600] Moustakas C. (1990). Heuristic research: design, methodology, and applications. Newbury Park, CA: Sage.

[ref650] Moustakas C. (1994). Phenomenological research methods. Thousand Oaks, CA: Sage.

[ref30] Newman R. Rashid M. A. (2025). There is no place like home: a Meta-ethnography exploring the experiences of international medical graduates following return migration to their home country. Clin. Teach. 22:e70188. doi: 10.1111/tct.70188, 40874501 PMC12392376

[ref31] Noblit G. W. Hare R. D. (1988). Meta-Ethnography: Synthesizing Qualitative Studies. Thousand Oaks: Sage.

[ref32] Oakley L. Kinmond K. Humphreys J. (2018). Spiritual abuse in Christian faith settings: definition, policy and practice guidance. J. Adult Prot. 20, 144–154. doi: 10.1108/jap-03-2018-0005

[ref33] Page M. J. McKenzie J. E. Bossuyt P. M. Boutron I. Hoffmann T. C. Mulrow C. D. . (2021). The PRISMA 2020 statement: an updated guideline for reporting systematic reviews. BMJ 372:n71. 33782057 10.1136/bmj.n71 PMC8005924

[ref34] Pargament K. I. (2011). Spiritually Integrated Psychotherapy: Understanding and Addressing the Sacred. New York: Guilford Press.

[ref35] Pargament K. I. Exline J. J. (2022). Working with Spiritual Struggles in Psychotherapy: From Research to Practice. New York: Guilford Press.

[ref550] Pargament K. I. Mahoney A. (2005). Sacred matters: sanctification as a vital topic for the psychology of religion. Int. J. Psychol. Relig. 15, 179–198. doi: 10.1207/s15327582ijpr1503_1, 40874501

[ref36] Pargament K. I. Smith B. W. Koenig H. G. Perez L. (1998). Patterns of positive and negative religious coping with major life stressors. J. Sci. Study Relig. 37, 710–724. doi: 10.2307/1388152

[ref37] Picciotto G. Fox J. Neto F. (2017). A phenomenology of spiritual bypass: causes, consequences, and implications. J. Spiritual. Ment. Health 20, 333–354. doi: 10.1080/19349637.2017.1417756

[ref38] Ratts M. J. Singh S. A. Nassar-McMillan S. Butler S. K. McCullough J. R. (2016). Multicultural and social justice counseling competencies: guidelines for the counseling profession. J. Multicult. Couns. Dev. 44, 28–48. doi: 10.1002/jmcd.12035

[ref39] Rice S. M. (2023). Spiritual Bypass among African Americans in Addiction Recovery Programs: An Explanatory Sequential Mixed Methods Study. [Doctoral Dissertation]. St. Davids, PA: Eastern University.

[ref40] Sue D. W. Arredondo P. McDavis R. J. (1992). Multicultural counseling competencies and standards: a call to the profession. J. Couns. Dev. 70, 477–486. doi: 10.1002/j.1556-6676.1992.tb01642.x

[ref41] Sulmasy D. P. (2002). A biopsychosocial-spiritual model for the Care of Patients at the end of life. The Gerontologist 42, 24–33. doi: 10.1093/geront/42.suppl_3.24, 12415130

[ref42] Tong A. Flemming K. McInnes E. Oliver S. Craig J. (2012). Enhancing transparency in reporting the synthesis of qualitative research: ENTREQ. BMC Med. Res. Methodol. 12:181. doi: 10.1186/1471-2288-12-181, 23185978 PMC3552766

[ref44] Welwood J. (1984). Principles of inner work: psychological and spiritual. J. Transpers. Psychol. 16, 63–73.

[ref45] Welwood J. (2000). Toward a Psychology of Awakening: Buddhism, Psychotherapy, and the Path of Personal and Spiritual Transformation. Boston: Shambhala.

[ref46] Wilber K. (2000). Integral Psychology: Consciousness, Spirit, Psychology, Therapy. Boston: Shambhala.

[ref48] Zimmer L. (2006). Qualitative Meta-synthesis: a question of dialoguing with texts. J. Adv. Nurs. 53, 311–318. doi: 10.1111/j.1365-2648.2006.03721.x, 16441536

